# Witness Response at Acute Onset of Stroke: A Qualitative Theory-Guided Study

**DOI:** 10.1371/journal.pone.0039852

**Published:** 2012-07-20

**Authors:** Stephan U. Dombrowski, Falko F. Sniehotta, Joan Mackintosh, Martin White, Helen Rodgers, Richard G. Thomson, Madeleine J. Murtagh, Gary A. Ford, Martin P. Eccles, Vera Araujo-Soares

**Affiliations:** 1 Institute of Health & Society, Newcastle University, Baddiley-Clark Building, Newcastle upon Tyne, United Kingdom; 2 Institute for Ageing & Health, Newcastle University, Newcastle upon Tyne, United Kingdom; 3 Department of Health Sciences, University of Leicester, Leicester, United Kingdom; 4 Acute Stroke Service, Royal Victoria Infirmary, Newcastle upon Tyne Hospitals National Health Service Foundation Trust, Newcastle upon Tyne, United Kingdom; Cardiff University, United Kingdom

## Abstract

**Background:**

Delay in calling emergency medical services following stroke limits access to early treatment that can reduce disability. Emergency medical services contact is mostly initiated by stroke witnesses (often relatives), rather than stroke patients. This study explored appraisal and behavioural factors that are potentially important in influencing witness behaviour in response to stroke.

**Methods and Findings:**

Semi-structured interviews with 26 stroke witnesses were transcribed and theory-guided content analysed was undertaken based on the Common Sense Self-Regulation Model (appraisal processes) and Theory Domains Framework (behavioural determinants). Response behaviours were often influenced by heuristics-guided appraisal (i.e. mental rules of thumb). Some witnesses described their responses to the situation as ‘automatic’ and ‘instinctive’, rather than products of deliberation. Potential behavioural influences included: environmental context and resources (e.g. time of day), social influence (e.g. prompts from patients) and beliefs about consequences (e.g. 999 accesses rapid help). Findings are based on retrospective accounts and need further verification in prospective studies.

**Conclusions:**

Witnesses play a key role in patient access to emergency medical services. Factors that potentially influence witnesses’ responses to stroke were identified and could inform behavioural interventions and future research. Interventions might benefit from linking automatic/instinctive threat perceptions with deliberate appraisal of stroke symptoms, prompting action to call emergency medical services.

## Introduction

Delay in calling emergency medical services (EMS) following stroke onset is an important factor that prevents patients accessing hyperacute stroke care and thrombolytic therapy, an intervention which can have a beneficial impact on patient outcome [1]. Delay times range from 3 to 6 hours [2] reducing the chances of optimal recovery for the majority of stroke patients. EMS are typically contacted by witnesses of stroke, not stroke patients [3]. EMS phone calls for stroke are initiated by 2–7% of patients, with remaining contacts made by witnesses including family members (∼41–75%), friends (∼4–20%), or medical/care personnel (∼13–42%) [3]–[7]. Nevertheless, most research to date has focused on the patient rather than witness factors that determine response behaviour following stroke. However, different factors might influence witnesses’ compared with patients’ responses. It has been suggested that family members and friends should be targeted for interventions as they are likely to act on behalf of the patient when stroke occurs [2]. Past public education campaigns [8], [9] included stroke witnesses as a target population, but overall most interventions have been found to be only minimally effective [2], [10], [11]. Such interventions would benefit from a sound understanding of factors that influence witnesses’ response behaviour to stroke.

Despite the advantages of theory-based research and interventions, [12] the field of help-seeking behaviour often lacks rigorous methods [13] including an explicit theoretical basis [14], [15] and systematic intervention development [10]. Descriptive theories of the patient process in seeking medical care outline a number of distinct stages, [16]–[18] including one stage of situational appraisal where a health threat is detected (appraisal stage) and another stage where response behaviours are selected (behavioural stage). Our study explored factors and processes that might influence witness responses to stroke by examining witnesses’ interviews of their stroke experience using two theoretical frameworks that provide explanatory accounts of appraisal and behavioural factors.

First, the Common Sense Self-Regulation Model [19] was selected as it provides a detailed explanatory account of the processes involved in making sense of a health threat. According to the model, individuals faced with a health threat form a mental representation which reflects the individuals’ understanding of the illness. These illness representations include aspects of the illness such as its label (identity), its time-course (timeline), its effects on the person (consequences*)*, its controllability (personal control) and its cause. The Common Sense Self-Regulation Model has previously informed research on health care seeking behaviour [20]. Second, the Theory Domains Framework [21] was selected as it provides a broad and inclusive evidence-based account of all relevant factors outlined in current theories of behaviour. This framework comprises 12 domains of theory-based explanations for behaviour resulting from a comprehensive review of behavioural theories and an expert consensus approach grouping theoretical constructs by commonality.

## Methods

### Design

Semi-structured interview study with a purposive sample to represent the range of response times from the onset of stroke symptoms to health services contact (i.e. >1 hour and <1 hour following stroke onset), based on self-report verified with medical staff in cases of uncertainty. Health service contact included telephoning EMS (i.e. dialling 999 [999 is UK equivalent to USA 911, and many other countries]), telephoning the primary care physician office (i.e. general practice surgery) and presenting to the emergency department (ED). Witnesses were recruited between April 2009 and January 2010 from three stroke units in north east England. Individuals reported by patients as having initiated health services contact leading to hospital admission on their behalf were initially approached by stroke research nurses, either on hospital site when accompanying patients, or via phone. Details of those interested were passed on to the research team.

### Procedure

Semi-structured interviews using a topic guide were conducted to explore witnesses’ reasons for responding to acute stroke. Interviews covered appraisal (“*What did you think was happening/had happened*?”), behavioural (“*What did you decide to do about the symptoms*?”), cognitive (“*What were important factors in making that decision*?”), and emotional (“*What were your main concerns/worries at the time*?”) factors. Interviews took place in witnesses’ homes and were audio-recorded, transcribed and anonymised. This study received ethical approval from the National Health Service (NHS) Sunderland Research Ethics Committee (REC08/H0904/104). All participants provided written, informed consent prior to participation.

### Analysis

Theory-guided content analysis to examine the appraisal and behavioural factors relevant for stroke responses was based on the two theoretical frameworks. Appraisal factor codings were based on the Common Sense Self-Regulation Model (CS-SRM) [19]. The concepts derived from this model for the purpose of this study are illness representations, including: ‘symptoms/consequences’ (i.e. a descriptive account of the perceived symptoms or consequences of symptoms), ‘identity/cause’ (i.e. an interpreting account of the perceived symptoms in terms of the underlying condition or cause), ‘cure/control’ (i.e. possibility of curing/controlling symptoms or illness), and ‘timeline’ (i.e. duration or length of symptoms or illness). In addition the Common Sense Self-Regulation Model includes the concept of heuristics, defined as strategies and mental rules of thumb used to respond to illness indicators; these were coded [22], [23].

Behavioural factor codings were based on the Theory Domains Framework [21], which included the following domains: knowledge; skills; social role & identity; beliefs about capabilities; beliefs about consequences; motivation and goals; decision processes; environmental context; social influences; emotion; behavioural regulation; and past behaviour.

Codings were made in NVivo9 for (a) the appraisal process, and (b) behavioural factors in a two-step process. in step 1, sections of the transcript were allocated to theoretical variables as outlined above. Transcript sections could be allocated to several constructs if these embraced multiple aspects or meanings. In step 2, theoretical variables reported to influence response behaviours were selected. Response behaviour was defined as any overt and observable behavioural response to witnessing stroke. Behavioural influence was determined by direct verbal reports (e.g. ‘…because of factor X I did behaviour Y’), coincidence (e.g. participants reporting a variable were more likely to engage in a particular response), or logic (e.g. perceptions logically prevented a certain response). Construct allocation and links to response behaviour were coded by one author (SUD) and double checked by a second author (VAS). Disagreements (12% and 7% of step 1 and 2 codings respectively) were resolved in discussion with a third author (FFS).

## Results

Twenty-six stroke witnesses (20 female) were recruited ≤14 days post stroke with the help of nurses at three participating stroke units in North-East England. Witnesses’ relationships to the patients were: wife/husband (*n* = 14), son/daughter (*n* = 9), granddaughter (*n* = 1), formal care-giver (*n* = 1) and acquaintance (*n* = 1). All strokes occurred in the patient’s home, except one that occurred in a supermarket. Most contacts with EMS (*n* = 13) and some to primary care physician offices (*n* = 2) were made within 1 hour of symptom onset. For those who responded after 1 hour, contact included EMS calls (n = 7), primary care physician (*n* = 3) and ED visits (*n* = 1). All variables reported in the next section were identified as influencing witness’ response behaviours.

### Appraisal Processes

#### Symptoms/Consequences

‘Symptoms/consequences’ refers to witnesses’ perceptions of the symptoms and consequences of a patient’s stroke. All symptoms/consequences perceived by witnesses were observable and influenced some witness responses (“…*his face was funny but all down his right side [he] couldn’t move it. So I […] phoned the doctor*”, W02, wife, GP<1 h [Abbreviations following quotes indicate: (a) witness number, (b) relation to stroke patient, (c) time between onset of symptoms and type of health service contacted.]), albeit most witnesses reported additional influences (outlined below). Perceptions of symptoms/consequences also guided how witnesses responded to stroke (*“I didn’t phone up and say: ‘My Gran’s had a stroke’ I just said: ‘My Gran isn’t moving’*.*”* W24, granddaughter, 999<1 h).

#### Identity/Cause

‘Identity/cause’ is the label (e.g. stroke) or perceived cause of observed symptoms. One third of witnesses reported successfully recognising the identity/cause of observed symptoms as stroke, often instantaneously, which frequently led to an immediate response (“*I knew straight away that she had had a stroke so I phoned straight away the ambulance*” W03, daughter, 999<1 h). One third suspected stroke and a further third failed to recognise stroke altogether. Failure to recognise stroke tended to be associated with the reporting of alternative explanations such as ‘shock’ or ‘food poisoning’, often accompanied by more varied and delayed response patterns.

#### Cure/Control

‘Cure/control’ represents witnesses’ perceptions about the ability to cure or control stroke symptoms. Witnesses often perceived a lack of personal capability to contribute to cure/control patients’ symptoms, which commonly led to a help-seeking response, indicating the perceived ability of health professionals to be able to cure/control symptoms (*“I mean I couldn’t cope with him the way he was* [unilateral weakness and speech problems] *and I said: ‘[…] I am phoning the doctor’”* W02, wife, 999<1 h). Lack of cure/control was often expressed in terms of fear of death, but none of the witnesses reported perceiving death as inevitable.

#### Heuristics

Many witnesses used heuristics (i.e. mental rules of thumb) to evaluate the observed symptoms. Heuristic-guided information processing seemed to facilitate both the recognition of a health threat (i.e. appraisal stage) and subsequent responses (i.e. behavioural stage). The ‘discrepancy heuristic’ includes a comparison process between the witness’ expectations and actual perceptions of the patient, for example based on expected and perceived health status. Large discrepancies typically lead to the detection of ‘something being wrong’ (*“*…*she wasn’t her normal self”,* W25, daughter, EMS<1 h), in some cases immediately, and was often coupled with an instant response (“*I could tell straight away […] that there was something wrong, as soon as I came in I said ‘I’ll phone an ambulance’*”, W09, wife, EMS<1 h). In addition, expectation-perception discrepancies could be detected in terms of behaviour, where witnesses reported patients behaving untypically (“*…if he tells me to ring, it must be serious*” W26, wife, EMS>1 h), prompted the inference of a problem, and in some cases a swift response. Further heuristics influencing responses were the ‘comparison heuristic’ including self-comparison (*“… we both had it* [food poisoning] *I thought well I’ve got it […] so we put it off until the next day”* W04, wife, GP>1 h) and stroke prototype comparison (*“…she doesn’t have high blood pressure; she eats a very healthy diet, […] she doesn’t smoke, […]. So yeah, it came as a surprise to me.”* W21, husband, GP>1 h).

### Behavioural Determinants of Stroke Responses

#### Knowledge

Here ‘knowledge’ refers to symptom knowledge (i.e. knowing the symptoms indicating stroke), response knowledge (i.e. knowing how to respond to stroke) and treatment knowledge (i.e. knowing the available treatments for stroke). Symptom knowledge varied greatly and, when present, could lead to stroke recognition and a rapid response (*“…when I got closer I realised she had had a stroke because her mouth had dropped and she couldn’t get her words out so straight away I phoned 999”*, W03, daughter, 999<1 h). Most witnesses who recognised stroke also contacted EMS within an hour (*“…I thought if dad has had a stroke I know that within a certain length of time you’ve got to get medical assistance”*, W22, daughter, 999<1 h), with those only suspecting stroke often delaying an EMS response. Treatment knowledge for acute stroke was generally absent regardless of symptom and response knowledge levels.

Limitations of knowledge as a factor influencing response behaviours emerged from witnesses accounts. A lack of stroke knowledge could co-occur with an appropriate response (*“I don’t know very much about strokes, but I knew that he needed help”* W17, wife, 999<1 h). Knowledge could also be perceived as irrelevant if the situation itself warranted an emergency response, independent of stroke recognition *(“…it seemed obvious to me, there was definitely something wrong, whether it was a stroke or not I had to get the ambulance”* W10, husband, 999>1 h [Response coded as >1 h as stroke occurred during the night, witness responded immediately following encounter of symptoms.]). Knowledge misconceptions were often reported to delay immediate EMS responses, including misconceptions on symptom placement (*“…[for] a full stroke the mouth would probably be down”* W23, son, 999>1 h), recognition (*“I recognised the TV* [advertised symptoms and] *said: ‘Can you put your arms up?’ … He says: ‘I’m putting my arms up’. I’m thinking: ‘Well it cannot be a stroke’”* W04, wife, GP>1 h), or symptom patterns (“…*the eye goes, the mouth goes, the arm goes limp, - that’s when you do something*” W06, daughter, GP>1 h). Misconceptions of the EMS remit also seemed to prevent swift service engagement (*“[EMS are] obviously for people [that have] been rushed in with car accidents, or heart attacks”,* W05, daughter, 999>1 h).

#### Social influence

‘Social influence’ incorporates social interactions influencing stroke responses and some witnesses reported responding to stroke following patient prompting, (*“…he just said: ‘Help me’, and that’s when I called for the ambulance”* W7, son, 999<1 h). Other prompts from health care professionals were mostly followed regardless of whether EMS contact was recommended (*“[The GP] says: ‘Put the phone down, dial 999’ […], so I did that”*, W02, wife, GP<1 h) or not (*“…[The GP] just said to take him straight up to A&E”* W11, wife, A&E>1 h). In some cases behavioural decisions were negotiated between witness and patient (“…*[the patient said] ‘Oh don’t bother with the ambulance […] I’ll be okay’, I wasn’t having none of that, enough is enough”*, W10, husband, EMS>1 h) and several witnesses took patient wishes into consideration when making the decision (*“My Mum doesn’t like a fuss made and she was like: ‘Don’t you dare call the doctor or an ambulance’. So I rang NHS Direct”* W19, daughter, 999>1 h). Perceived norms for accessing NHS help also influenced responses in a few cases *(“I rang the health line which they did tell you to ring first before you dial 999”* W15, wife, 999<1 h).

#### Beliefs about consequences

‘Beliefs about consequences’ are witnesses’ expectations of what would follow if a particular response was performed. Beliefs about consequences associated with a swift response included: negative outcome expectations in the absence of immediate medical care (*“…the reason I rang for the ambulance to get her into hospital was I thought: ‘Well if we leave it, it might get worse’”* W05, daughter, 999<1 h), response speed (“*I just picked up the phone, 999, it was quick, seconds. Just for quickness*”, W12, wife, 999<1 h), or a combination of the two (*“I thought he needed the help straight away rather than ring the doctors and whatever I just thought I’ve got to do that* [dial 999]*”*, W09, wife, 999<1 h). Beliefs about consequences of a response associated with delay were: confirmation of stroke suspicion (*“I thought it was possible that it was a stroke, but that’s why I phone NHS direct just to confirm”* W16, wife, 999<1 h), obtaining advice (*“What I did was I said: ‘I will phone the surgery and get advice’, I thought maybe from the nurse”* W11, wife, A&E>1 h), or inconveniencing the health service (*“they* [EMS] *are such a busy service and you think: ‘If I can help by getting my Mum there and not have to trouble them, they are for somebody who hasn’t got transport or hasn’t got family near’”* W06, daughter, GP>1 h).

#### Decision processes

‘Decision process’ is the process through which witnesses arrived at a response following witnessing stroke. The behavioural decision-making process included automatic/instinctive as well as reflective/deliberative decision making elements and some evidence of both instinctive and deliberative decision-making processes influencing response behaviours emerged, which could lead to swift EMS contact (*“I think I made [the decision to call EMS] straight away. I must admit I did think ring the doctor and then I thought: ‘No I’m going to ring an ambulance*’*”* W09, wife, 999<1 h) but not in all cases *(“… my first instinct was that it’s been a stroke but I couldn’t have dialled 999 because she would have lost her temper”*, W19, daughter, 999>1 h).

#### Motivation and goals

‘Motivation and goals’ are witnesses’ reasons for performing a particular response. A frequently reported motivation for a particular response was the goal of getting help quickly, which almost always associated with swift EMS contact (*“…the one thing that I wanted to do was just get somebody here quick”*, W22, daughter, 999<1 h). There was some suggestion that witnesses went through a selection process where response options were considered and selected.

#### Environmental context

‘Environmental context’ includes influences of witnesses’ physical context on their response behaviour. Environmental context influencing witnesses’ response included time of day, mostly in cases where GP surgery contact was initially contemplated. If the stroke was detected past/before surgery opening hours some witnesses contacted EMS instead *(“I then said: ‘I am going to get the paramedics’, because it was at night”* W08, acquaintance, 999>1 h), but for most respondents this was associated with initial non-EMS contacts (*“I thought it was too late to phone them so I thought I’ll phone NHS direct”* W16, wife, 999<1 h).

#### Past behavior

Past behaviour (*“I called 999 that time as well … with her having the two previous strokes”* W05, daughter, 999<1 h) was reported by some witnesses as reasons for responses. Some reported negative past experiences as a reason for a particular responses (“*…I thought by the time I go through all this you know rigmarole with the other one I’m just as quick dialling 999*”, wife, 999<1 h).

#### Emotion

‘Emotion’ refers to witnesses’ affective reactions following the witnessing of stroke. Affective reactions following the witnessing of stroke were frequently reported as by-products of witnesses’ appraisal and response processes, and seemed to influence speed and performance (“*…it was a shock but I was sort of in a daze really*”, W09, wife, EMS<1 h), or (recall of) the decision making process (“*…well just panic. I don’t know what was going through my mind because […] I really don’t know what went through my mind*”, W11, wife, A&E>1 h).

## Discussion

This study outlines multiple interrelated factors likely to influence witnesses’ responses to stroke. Different processes and determinants may result in similar response behaviours and some influence of stroke appraisal and behavioural factors may operate out of immediate awareness.

Heuristics seemed to guide information processing, often regardless of symptom recognition, sometimes contributing to an immediate response. As acute stroke is often a complex presentation, heuristics might play a crucial role in adaptively guiding witness’ appraisal and response processes by ignoring redundant information (e.g. loss of speech requires immediate medical attention regardless of other symptoms). Complexity-reducing mental shortcuts to behaviour might be used in rapid-onset high-threat conditions such as stroke, as deliberation of all relevant stimuli could lead to more harmful outcomes. Heuristics have been shown to result in superior behavioural responses in a variety of situations as compared to more complex processes [23] and may partly explain findings in the delay literature, such as that less severe strokes are associated with longer delay [24] and differential delay patterns for different stroke strategies [25].

Equal proportions of witnesses identified, suspected or failed to recognise stroke (one-third each respectively). Those failing to recognise stroke often formed alternative conclusions about symptom identities/causes. Once a health threat was detected, increasingly elaborate mental representations of the patients’ health condition were formed. Illness representations seemed to influence responses, with those typically not associated with an emergency (e.g. food poisoning) potentially causing delays compared to illness representations typically associated with emergency (e.g. heart attack). Witnessing stroke is likely to lead to a rapid instinctive appraisal and responses followed by more elaborate processes taking additional factors into consideration.

This study might help to explain other common findings in the stroke delay literature, such as the gap between stroke knowledge and response behaviour [24], [26]. Knowledge appeared to be beneficial, but neither necessary nor sufficient factor to influence appropriate responses for the following reasons:

1. Knowledge of symptoms might not lead to stroke recognition (e.g. a witness has factual knowledge of all symptoms but fails to match knowledge with an occurring stroke in the moment);
2. Stroke (or other serious illness) recognition might occur even if witnesses have poor stroke knowledge (e.g. a witness only knows a limited number of symptoms but encounters one of these, recognises stroke and responds adequately);
3. Following stroke recognition, the appropriate response might be unknown (e.g. a witness recognizes stroke, but thinks it is right to contact the primary care physician instead of EMS);
4. Knowledge effects might be masked by other factors with potentially stronger influence on behaviour (e.g. a witness recognizes stroke and the need to contact EMS, but delays to avoid inconveniencing busy services).

Further research needs to explore the processes through which knowledge influences responses, using explanatory frameworks such as the Common Sense Self Regulation Model [19]. Descriptive accounts of the varying relationship between knowledge and response can only be advanced by exploring how such effects are being generated.

Social influence seemed to play a key role. Witnesses typically engaged in conversations with patients, other witnesses and health care professionals, all of which appeared to influence behaviour with the potential to both increase or decrease delay. Some witness/patient interactions included shared decision-making elements with most patients reluctant to engage EMS, suggesting that compared to patients, witnesses’ threshold for initiating an emergency response might be lower.

Our findings suggest several factors that offer promising opportunities for the development of delay-reducing interventions, some of which are discussed below. Key factors to target are heuristics that can lead to swift behavioural performance. Behavioural interventions might use unspecific and automatic perceptions of ‘something is wrong’ as triggers for an immediate and more focused appraisal for stroke using the FAST check list [8]. This could potentially accelerate the initiation of the appropriate response by the witness.

Furthermore, as stroke identification is complex, suspicion of stroke could be linked with immediate contact of EMS. Evidence suggests witnesses are mostly correct in their diagnosis when suspecting stroke [3]. Action plans [27] to pre-specify calling EMS upon suspecting stroke could decrease delay. Additionally, coping planning [27] for probable behavioural tendencies (e.g. contacting peers or non-EMS professionals) could be counteracted by planning to immediately contact EMS on suspicion of stroke, thereby ignoring competing behavioural options.

The social elements of witnessing stroke could be used by at-risk patients to collaboratively plan [28] response patterns in the event of stroke. Pre-specifying perceived stroke symptoms as triggers for social interaction to immediately call EMS could interfere with time consuming deliberation processes. Further intervention targets might constitute strengthening of the link between contacting EMS and positive outcomes in the short (e.g. quick help) and long-term (e.g. better recovery chances) through accessing effective treatments.

The strengths of this paper are the focus on the key population of stroke witnesses and the use of psychological theory to explore factors relevant for determining response behaviours. The limitations of this research are that the relative importance of identified factors and their likely interactions cannot be determined using qualitative methods. Moreover, the use of different theoretical frameworks, might have uncovered additional important factors not captured within the models employed in the current analysis. Lastly, retrospective accounts of witness’ experience are prone to recall bias and the sample obtained for this study might not be representative of the population of stroke witnesses.

Future research should include both explanatory and descriptive frameworks to understand the process underlying delay. Carefully unpacking witnesses’ behavioural response sequences when encountering stroke and determining the factors important for different response behaviours would further increase our understanding. In addition, interactions between witnesses and patients need to be assessed. The differences in processes and determining factors between witnesses and patients need exploration to understand common and distinct targets for appropriate behavioural interventions. Lastly, given the overlap of stroke symptoms with other health conditions and the significant risk of co-morbidity in patients at-risk of stroke, research needs to determine whether EMS responses should be advocated at the symptom level (i.e. symptom X  =  seek care Z) or at the disease level (i.e. symptom X indicates disease Y  =  seek care Z) [14]. Although seemingly similar, the two levels of focus would have implications for stroke interventions and impact on how these could be dovetailed with those for other conditions.

Customary calls for more education and awareness of stroke should be supplemented with elaborations on the specific factors that interventions should actively target. Focusing on the key population of witnesses, the current study advances our cumulative understanding of delay between stroke symptom onset and calling EMS and suggests specific targets to maximise stroke response efficiency.
